# Global patterns and trends in kidney cancer incidence and mortality

**DOI:** 10.1002/ijc.70349

**Published:** 2026-01-29

**Authors:** Anton Barchuk, Jerome Vignat, Kari A. O. Tikkinen, Ahmedin Jemal, Freddie Bray, Ariana Znaor

**Affiliations:** ^1^ Cancer Surveillance Branch The International Agency for Research on Cancer Lyon France; ^2^ Department of Medical Informatics Erasmus MC Rotterdam The Netherlands; ^3^ Faculty of Medicine University of Helsinki Helsinki Finland; ^4^ Department of Health Research Methods, Evidence, and Impact McMaster University Hamilton Ontario Canada; ^5^ Department of Urology University of Helsinki and Helsinki University Hospital Helsinki Finland; ^6^ Department of Surgery Päijät‐Häme Central Hospital Lahti Finland; ^7^ Surveillance and Health Equity Science American Cancer Society Atlanta Georgia USA

**Keywords:** incidence, kidney cancer, mortality, trends

## Abstract

This study provides an update on global patterns and trends in kidney cancer incidence and mortality. We used the most recent GLOBOCAN estimates, based on the best available data sources, including population‐based cancer registries (PBCRs), to compare incidence and mortality in 185 countries or territories in 2022 and to assess time trends based on recorded PBCR and vital statistics data in 71 countries. Incidence age‐standardised rates (ASRs) varied 10‐fold across UN regions and 20‐fold at the country level in 2022. Kidney cancer ASRs were consistently higher in males than females and ranged from 1.6 per 100,000 in low Human Development Index (HDI) countries to 12.6 in very high HDI countries among men and from 1.1 to 5.9 among women. The highest ASRs in males were in Belarus (22.9), Uruguay (20.5), and Latvia (19.2), and in females in Latvia (9.5), Uruguay (8.7), USA (8.7). While the patterns were similar for mortality, variations were less pronounced. The mortality‐to‐incidence ratio was lowest in Oceania (0.2) and highest in Africa (0.7). Over the past 15 years, incidence ASRs have tended to increase or stabilise in most European countries, Northern America and Oceania, but increased in Asia and Latin America. Mortality ASRs decreased in most countries, but increased in Portugal, Romania, Moldova, the Philippines, Malaysia and in 9 of 14 countries in Latin America. Regional variations in incidence call for a greater focus on risk factors amenable to prevention, coupled with an assessment of the role of diagnostics. The varied mortality patterns indicate present treatment inequalities.

## What's New?

Decreasing mortality from kidney cancer in developed countries contrasts with persistently high or rising mortality in less‐developed regions. Factors underlying this widening gap remain incompletely understood. Using GLOBOCAN 2022 data, the present study examined patterns in kidney cancer incidence and mortality worldwide and the relation of recent trends to known risk factors. Analyses reveal considerable variation in kidney cancer incidence, with differences partially explained by risk factors and access to diagnostics. Mortality is additionally linked to access to and availability of treatment. The findings suggest that primary prevention focused on modifiable risk factors could significantly reduce the global kidney cancer burden.

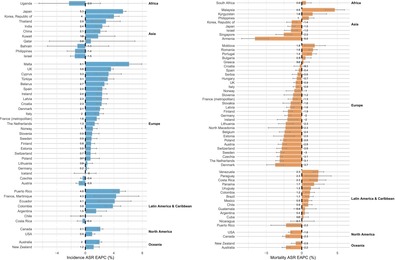

**Abbreviations**
ASR: age‐standardised incidence rates
CI: confidence interval
CI5: Cancer Incidence in Five Continents
EAPC: estimated annual percent change
HDI: Human Development Index
IAEA: International Atomic Energy Agency
IARC: International Agency for Research on Cancer
ICD‐10: International Classification of Diseases, 10th Revision
ICI: immune checkpoint inhibitors
M:I: mortality‐to‐incidence (ratios)
PBCR: population‐based cancer registries
TKI: tyrosine kinase inhibitor
UN: United Nations

## 1 INTRODUCTION

With an estimated 434,000 new cases and 155,700 deaths in 2022, kidney cancer was the 14th most common cancer and the 16th most common cause of cancer death globally.^1^ Rates tend to be elevated in high‐income countries, such as those in Europe, North America, and Oceania, compared to low‐income countries in regions like Africa and Asia.

While epidemiological studies have pointed to the marked geographic and temporal variations, kidney cancer incidence and mortality rates are consistently higher in men than in women.^2^, ^3^ Obesity, smoking, hypertension, and chronic kidney disease are established risk factors,^4^ with 19% of new cases globally attributed to obesity and a further 17% attributed to smoking.^5^ Other potential risk factors include urinary stone disease,^6^ diabetes,^7^ viral hepatitis,^8^ occupational exposure to trichloroethylene, cadmium and other substances,^9^ as well as the use of analgesics.^10^ Increased diagnostic intensity, possibly leading to overdiagnosis, likely contributes to elevated kidney cancer incidence rates, particularly in countries with high or very high Human Development Index (HDI) levels.^11^, ^12^ However, an analysis of global trends in 42 countries reported a recent stabilisation in incidence in high‐risk areas.^2^

Kidney cancer mortality has been decreasing in high and very high HDI countries but not in low and medium HDI countries based on the data available up to 2010, an observation ascribed to access to and availability of effective treatment.^2^ Over the last two decades, kidney cancer management has evolved from the use of tyrosine kinase inhibitors (TKIs) to immune checkpoint inhibitors (ICIs) doublets and ICI/TKI combinations as preferred first‐line options for metastatic disease, and more recently the adoption of adjuvant PD‐1 inhibitor therapy for patients with high‐risk clear‐cell renal cell carcinoma.^13^, ^14^

This study aims to analyse and interpret the global variations and trends in kidney cancer incidence and mortality, with a view to assessing the prospects of control of the disease across the continuum.

## 2 METHODS

We extracted the estimated number of new diagnoses and deaths from kidney cancer (corresponding to the International Classification of Diseases, 10th Revision [ICD‐10] code C64) from the International Agency for Research on Cancer (IARC) GLOBOCAN 2022 database, which includes data for 36 cancer sites and 185 countries or territories by sex and 18 age groups (0–4, 5–9, …, 80–84, 85 and older). We extracted the corresponding population data for 2022 from the United Nations (UN) website.^15^ The data sources and hierarchy of methods used to compile cancer estimates are available elsewhere.^1^, ^16^ In brief, the GLOBOCAN estimates are assembled at the national level using the best available cancer incidence and mortality data sources within a given country. The methods used to derive the 2022^1^ estimates correspond to those used in estimates for previous years^17^; where applicable, priority is given to short‐term predictions and modelled mortality‐to‐incidence (M:I) ratios, while validity is dependent on the degree of representativeness and quality of the source information.^16^ Based on these sources, we computed the age‐standardised incidence rates (ASR) per 100,000 person‐years using the 1966 Segi‐Doll World standard population.^18^

To examine trends in incidence, we obtained the number of new kidney cancer diagnoses by selected population‐based registries and extracted the population at risk from successive volumes of Cancer Incidence in Five Continents (CI5), a compendium of high‐quality population‐based cancer registries (PBCR) at the national or subnational level.^19^ Where PBCR are not national (23 of 47 countries with incidence data available), the trends are based on one or more subnational PBCRs. We obtained equivalent mortality data based on national vital registration from the WHO mortality database for the same period from 57 countries. The data quality for country‐level causes of death is considered high for 47 countries and medium for 10 countries.^20^ To assess trends in recorded kidney cancer incidence and mortality ASR over time, we estimated the annual percent change (EAPC) and its 95% confidence interval (95% CI) for the most recent 15‐year period using a generalised linear model assuming a Gaussian distribution of the log‐transformed ASR. Analyses were conducted in R,^21^ version 4.4.3, and RStudio,^22^ mainly using the RCan package.^23^ We present the results by region, sub‐region, intermediary region, country or territory of the world defined by the UN,^15^ and the four‐tier HDI.^24^

## 3 RESULTS

### 3.1 Geographic variation of incidence and mortality rates in both genders

Kidney cancer incidence rates across UN regions varied 10‐fold among men in 2022, from 1.2 in Western Africa to 12.6 in North America. Among women, incidence rates ranged from 0.8 to 1.0 in Melanesia, Western and Middle Africa and South‐central Asia to 8.5 in Northern America (Table 1). Globally, the male‐to‐female ratio for incidence rates was 2.0, varying from 1.2 in Western Africa to 2.7 in Australia/New Zealand. Male‐to‐female ratio was 2.0 or above in Europe, America, Australia/New Zealand and between 1.0 and 2.0 in Asia (except South‐Eastern Asia) and Africa (Table 1).

**TABLE 1:** Absolute number of kidney cancer cases and deaths and incidence and mortality rates in UN regions and by Human Development Index level in 2022.

	Incidence					Mortality					M:I	
	Males		Females		M:F	Males		Females		M:F	Males	Females
Region	Cases	ASR	Cases	ASR		Deaths	ASR	Deaths	ASR			
Eastern Africa	2726	1.8	2532	1.3	1.4	1853	1.3	1671	0.9	1.4	0.7	0.7
Middle Africa	1009	1.5	879	1.0	1.5	679	1.1	570	0.8	1.4	0.7	0.8
Northern Africa	2752	2.5	2119	1.8	1.4	1372	1.3	1100	0.9	1.4	0.5	0.5
Southern Africa	868	3.3	591	1.8	1.8	361	1.5	263	0.8	1.9	0.5	0.4
Western Africa	1932	1.2	1721	1.0	1.2	1222	0.8	1082	0.7	1.1	0.7	0.7
Caribbean	950	3.5	636	2.3	1.5	412	1.4	260	0.8	1.8	0.4	0.3
Central America	4979	5.7	2604	2.5	2.3	2587	2.9	1368	1.3	2.2	0.5	0.5
South America	17,131	6.7	9627	3.2	2.1	7079	2.6	3972	1.2	2.2	0.4	0.4
Northern America	51,967	16.9	27,802	8.5	2.0	11,232	2.9	5745	1.2	2.4	0.2	0.1
Eastern Asia	66,292	4.8	34,650	2.4	2.0	22,178	1.4	10,729	0.6	2.3	0.3	0.3
All but China	18,986	8.9	8300	3.3	2.7	5788	2.0	3128	0.7	2.9	0.2	0.2
China	47,306	4.1	26,350	2.2	1.9	16,390	1.3	7601	0.5	2.6	0.3	0.2
South‐Eastern Asia	8176	2.3	4392	1.1	2.1	4340	1.3	2406	0.6	2.2	0.6	0.5
South‐Central Asia	17,014	1.7	10,217	1.0	1.7	9887	1.0	5630	0.6	1.7	0.6	0.6
All but India	5920	2.1	3831	1.3	1.6	3201	1.2	1852	0.7	1.7	0.6	0.5
India	11,094	1.6	6386	0.9	1.8	6686	1.0	3778	0.5	2.0	0.6	0.6
Western Asia	6769	5.0	3766	2.8	1.8	2774	2.2	1295	0.9	2.4	0.4	0.3
Eastern Europe	31,607	14.5	21,364	7.0	2.1	11,747	5.0	7168	1.8	2.8	0.3	0.3
Northern Europe	13,680	13.7	7742	6.8	2.0	4515	3.5	2722	1.6	2.2	0.3	0.2
Southern Europe	19,275	12.5	9515	5.1	2.5	6771	3.5	3515	1.3	2.7	0.3	0.3
Western Europe	27,090	13.0	15,448	6.2	2.1	10,304	3.9	5605	1.5	2.6	0.3	0.2
Australia/New Zealand	3486	13.7	1393	5.0	2.7	984	3.0	494	1.2	2.5	0.2	0.2
Melanesia	60	1.6	33	0.8	2.0	32	1.0	12	0.3	3.3	0.6	0.4
Micronesia/ Polynesia	37	5.5	9	1.3	4.2	14	2.0	3	0.3	6.7	0.4	0.2
HDI level												
Low HDI	6903	1.6	6028	1.3	1.2	4453	1.2	3728	0.8	1.5	0.8	0.6
Medium HDI	19,913	1.9	12,102	1.1	1.7	11,412	1.1	6841	0.6	1.8	0.6	0.5
High HDI	75,851	4.0	43,616	2.2	1.8	29,361	1.5	15,134	0.7	2.1	0.4	0.3
Very high HDI	175,071	12.6	95,259	5.9	2.1	55,090	3.3	29,896	1.3	2.5	0.3	0.2
World	277,800	5.9	157,040	3.0	2.0	100,343	2.0	55,610	0.9	2.2	0.3	0.3

Abbreviations: ASR(W), age‐standardised rate (world population standard [33]) per 100,000; HDI, Human Development Index; M:F, ratio of rates in males and female; M:I, mortality to incidence ratio.

**FIGURE 1:**
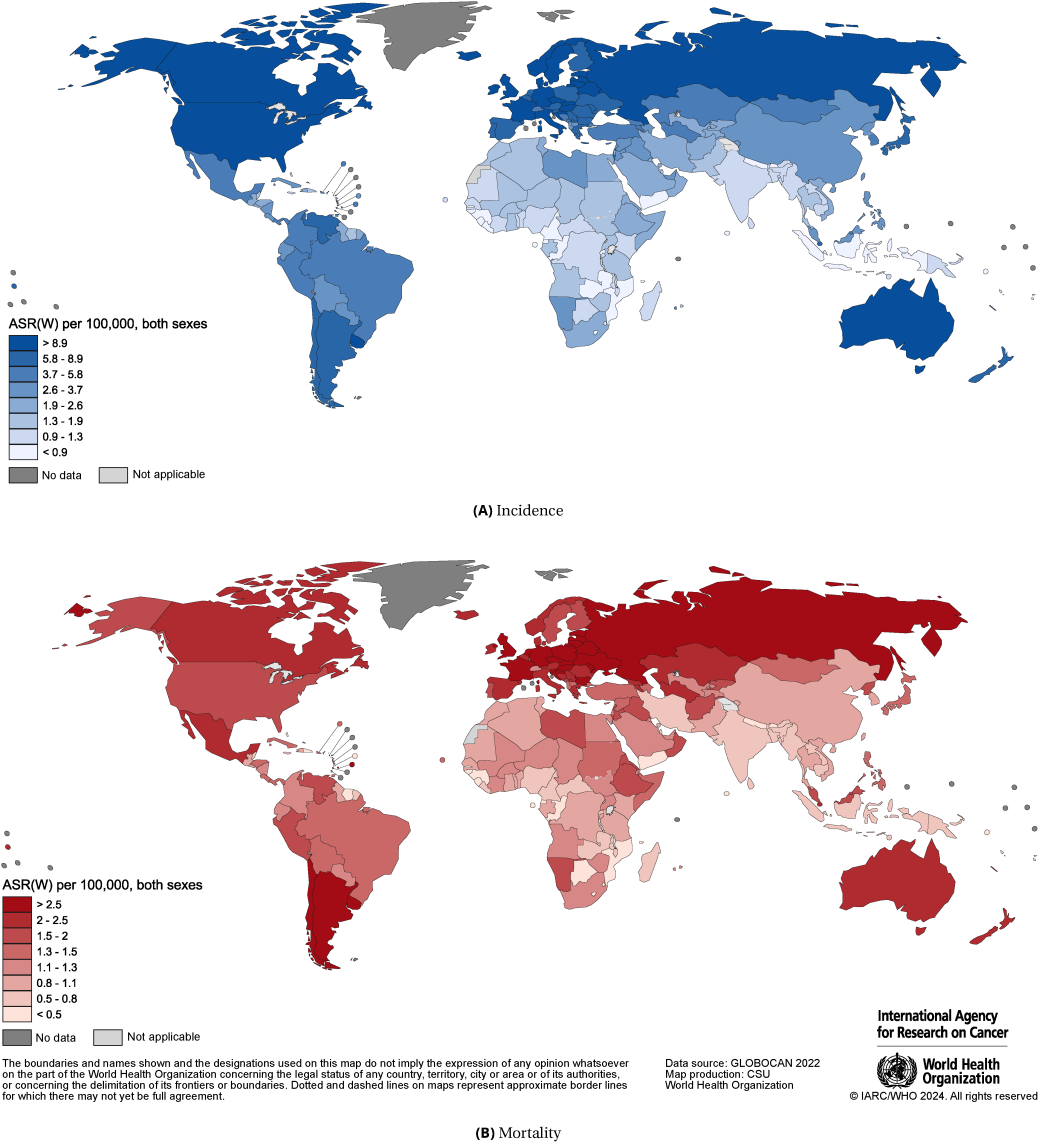
International variation in estimates of national age‐standardised kidney cancer incidence (A) and mortality (B) rates.

**FIGURE 2:**
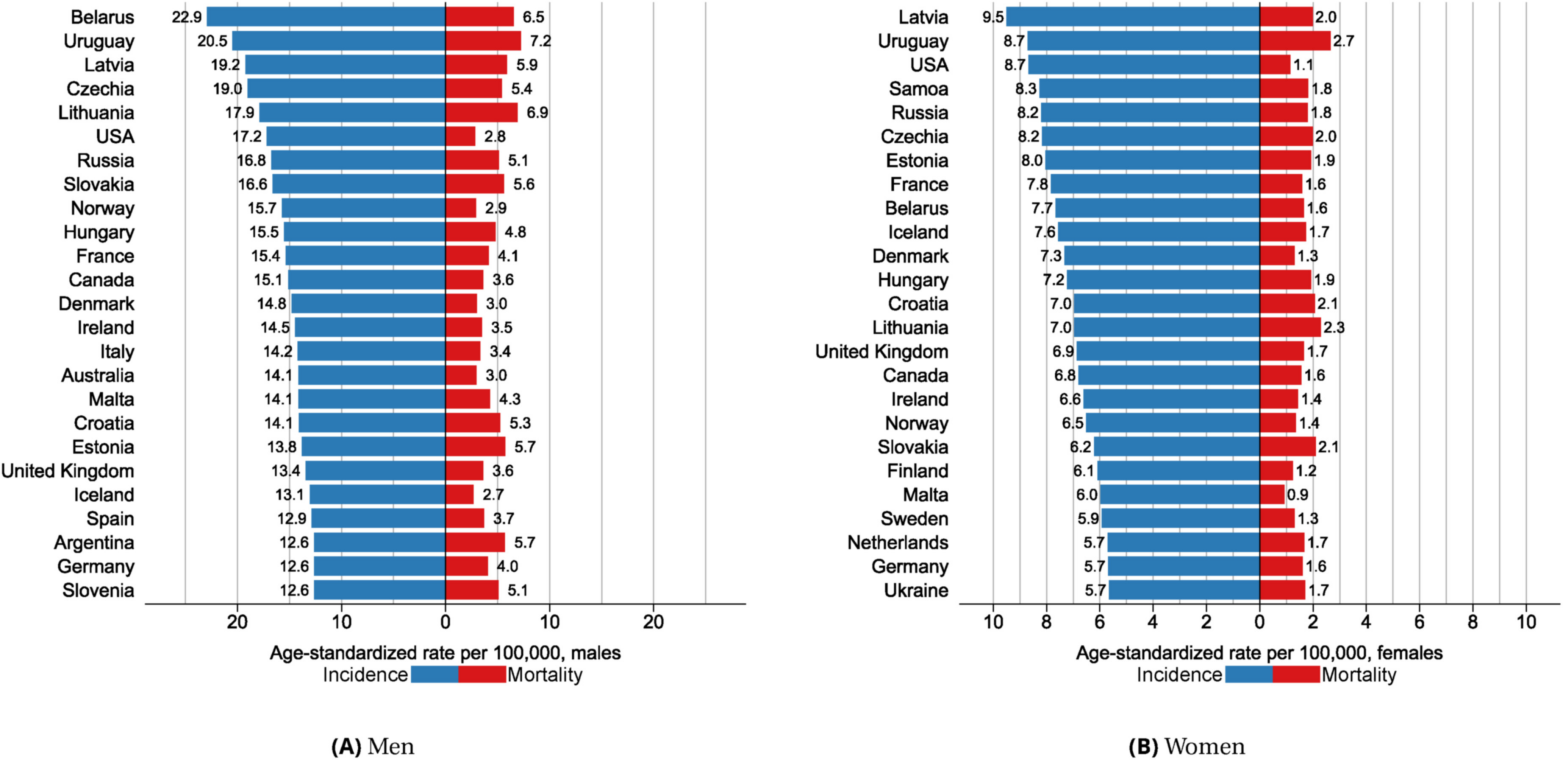
Top 25 countries (out of 185) with the highest kidney cancer incidence in men (A) and in women (B).

Overall, the incidence of kidney cancer was higher in North America, South America, Europe, and Australia/New Zealand and lower in Africa and Asia, with similar geographical patterns for men and women (Figure 1). However, the variation between countries within each region was also substantial (Figures 2, A1 and A2).

By HDI, incidence rates ranged from 1.6 in countries with low and medium HDI to 12.6 in countries with high HDI among men, and from 1.1 to 5.9 among women.

At the country level, we identified more than a 20‐fold variation in incidence, with the highest rates among men in Belarus (22.9), Uruguay (20.5), Latvia (19.2), Czechia (19.0), and Lithuania (17.9). Among women, the highest incidence was in Latvia (9.5), Uruguay (8.7), USA (8.7), Samoa (8.3), and Russia (8.2) (Figures 2, A1 and A2).

Although the geographic variations in kidney cancer mortality rates were less pronounced, patterns were similar to incidence. The highest mortality rates were in Eastern Europe (5.0 among men and 1.8 among women). As with incidence, mortality was higher in men in all regions (Table 1). The mortality‐to‐incidence ratio was lowest in Australia/New Zealand (0.2) and highest in African regions (0.7). At the country level, relatively high mortality rates were in several European countries (Belarus, Czechia, and Latvia) and South America (Uruguay and Argentina) (Figures 2, A1 and A2). The gradient in mortality rates by HDI was observed mainly in men, with rates ranging from 1.3 in low HDI to 3.3 in very high HDI countries (Table 1).

### 3.2 Trends in incidence and mortality rates

**FIGURE 3:**
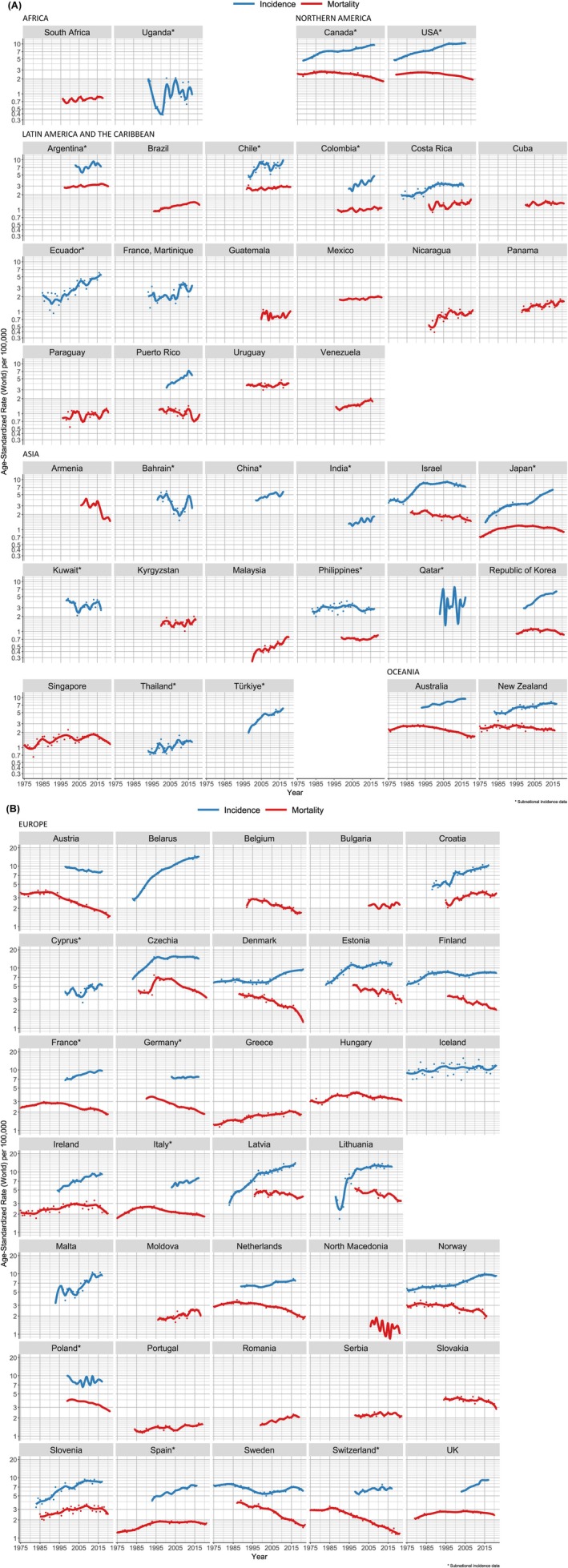
(A) Kidney cancer incidence (23 countries) and mortality (27 countries) trends in Africa, Northern America, Latin American & the Caribbean, and Oceania. (B) Kidney cancer incidence (24 countries) and mortality (30 countries) trends in Europe.

During the most recent 15‐year period, the direction and magnitude of trends in kidney cancer incidence and mortality rates over time varied markedly in different regions (Figure 3). While rates were higher in men than in women across all regions, as incidence and mortality trends were similar in men and women, we combined them for analysis (Figures A3–A6).

In most European countries, kidney cancer incidence was increasing or stable, with the largest increases over the last 15 years (estimated annual percent change EAPC >2%) detected in Italy, Denmark, Croatia, Latvia, Ireland, Spain, Belarus, Türkiye, Cyprus, the UK, and Malta. Mortality declined or remained stable in most countries, except Portugal, Romania, and Moldova. Mortality rates remained relatively stable in Croatia, Spain, Serbia, Greece, and Bulgaria (Figure 3B, Table A2). The greatest mortality declines (< −3% annually) were observed in Sweden, Denmark, the Netherlands, and Czechia.

**FIGURE 4:**
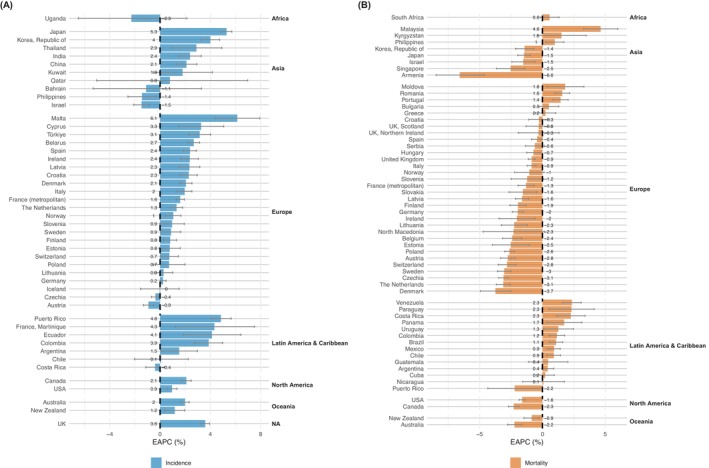
Estimated annual percent change in kidney cancer (A) incidence (47 countries) and (B) mortality (57 countries) rates in the last 15 years.

In Northern America, the incidence in both sexes increased (EAPC = 2.1%, 95% CI: 1.7; 2.5) per annum in Canada and 0.9% (0.6; 1.3) in the United States. The corresponding mortality trends were in decline in both countries (Figure 4, Table A1). While incidence rates have been increasing in Australia/New Zealand (2.0% (1.7; 2.3)) and 1.2% (0.4; 2.0), mortality rates have decreased (−2.2% (−2.8; −1.7) and −0.9% (−1.5; −0.2)) (Figure 4, Table A1).

In Asia, kidney cancer incidence has been increasing in Thailand, China, and India, but the largest significant increases were detected in Japan (5.2% (4.9; 5.7)) and the Republic of Korea (4.0% (3.3; 4.7)). Incidence rates declined in the Philippines and Israel but were relatively stable in other countries. Mortality rates declined in Japan, the Republic of Korea, Singapore, and Israel, but increased in Malaysia and the Philippines. Time trend data were available in only two African countries; incidence rates appear to be declining in Uganda, while mortality rates were increasing in South Africa, but neither estimate was significant (Figure 4, Table A1).

Finally, in all Latin American and Caribbean countries that reported incidence trends, mortality and incidence were stable or increased. Marked increases in incidence were seen in Colombia (3.9% (2.8; 5.0)), Ecuador (4.1% (1.9; 6.4)), Martinique (4.3% (1.2; 7.5)), and Puerto Rico (4.8% (4.0; 5.7)). Mortality rates have also been rising in nine out of 14 countries with available data across the region, with the largest increases in Costa Rica, Paraguay, and Venezuela (EAPC >2% in all three countries) (Figure 4, Table A1).

## 4 DISCUSSION

We found substantial inter‐ and intra‐regional variability in the incidence of kidney cancer, with elevated incidence rates seen in Northern America, Oceania, Latin America and the Caribbean, and Europe and relatively low rates in Asia and Africa. Kidney cancer incidence rates were twofold higher in men compared to women, and this pattern was similar in most populations. We also observed several distinct patterns in the direction and magnitude of incidence and mortality trends worldwide. In high and very high HDI countries, such as Western Europe, North America, and Oceania, as well as some Asian countries, incidence rates have been increasing or are rather stable, while mortality rates have been predominantly decreasing. In contrast, both incidence and mortality rates have been on the rise in Latin America and in some European countries (e.g., Portugal, Romania, and Moldova).

The existence of unidentified exposures has recently been postulated as an explanation of international differences in the incidence of kidney cancer.^25^ Although further studies are needed to identify new carcinogens, it is also important to critically assess all available evidence on documented risk factors, namely, smoking,^26^ obesity,^27^ hypertension,^28^ and chronic kidney disease.^29^ The global variation in the prevalence of these risk factors is substantial, and their combination and interaction can result in a variation in the true incidence of kidney cancer, which may be further modified by the ability of healthcare systems to identify those tumours through diagnostics.^12^

The distribution of kidney cancer risk factors varies substantially across regions. The prevalence of hypertension among adults aged 30–79 years is markedly higher in Eastern and Central Europe and several Latin American countries.^28^ Smoking prevalence among men is highest in Central and Eastern Europe.^26^ Similarly, the highest prevalence of kidney disease is observed in Eastern and Central Europe, with a high prevalence of kidney failure reported in parts of Latin America.^29^ Nevertheless, the rising burden of these risk factors in low‐ and middle‐income countries indicates a potential for increasing kidney cancer incidence in these regions.

The study also confirmed the marked variation in kidney cancer incidence by sex: men continue to have a higher risk of kidney cancer than women. The higher male‐to‐female ratios observed in high‐income regions likely reflect the overall higher incidence rates in those regions. Some studies explain this variation by factors that may differ between men and women, such as immune surveillance, genome surveillance mechanisms, and sex chromosomes^30^; others have also mentioned that height and body size might be contributory factors. In a large prospective cohort in the United Kingdom (the Million Women Study), the risk ratio for kidney cancer was 1.29, 95% CI (1.19–1.41) for every 10 cm increase in height.^31^ The US Vitamins And Lifestyle (VITAL) study results based on over 65,000 volunteers aged 50–76 years showed that almost 91% of the kidney cancer excess risk for men was explained by the height differences between men and women.^32^ The average height difference between the tallest and shortest populations is approximately 20 cm, almost twice that between men and women, which is 11–12 cm.^33^, ^34^ One could speculate that such differences contribute to the sex and regional differences in kidney cancer incidence rates. A greater proportion of taller people were born in countries in the Northern Hemisphere and Australia/New Zealand, corresponding to the highest kidney cancer incidence areas.^33^ From the prevention perspective, height should probably be viewed as a marker of another possible exposure. For instance, dairy consumption has been linked to an increased risk of kidney cancer in a case–control study conducted in Eastern European countries,^35^, and it could also be associated with greater adult height.^36^ However, the underlying causal pathways are likely more complex, involving multiple genetic, environmental and social determinants that together may contribute to an elevated kidney cancer risk in taller individuals.

The incidence rates are also relatively high in regions with a high prevalence of obesity, including North America, Eastern Europe, and Latin America.^27^ While obesity prevalence studies are routinely based on the body mass index, they tend to underrepresent height in favour of weight.^31^ Body surface area has been proposed as a better predictor of the risk of various types of cancer,^37^ hence more studies are needed to assess its utility as a risk marker for kidney cancer.

In addition to the distribution of risk factors, the variation in kidney cancer incidence should be viewed through the lens of available diagnostic resources. Overdiagnosis has been mentioned as one of the driving forces behind the increase in the incidence, reflecting the increased use of imaging from the 1980s.^38^ In Europe, the United States, Australia/New Zealand, and Latin America, a high prevalence of several risk factors is coupled with good access to medical diagnostics. According to IMAGINE, (the International Atomic Energy Agency Global Resources Database for Medical Imaging and Nuclear Medicine^39^), Australia/New Zealand, North America, Europe, Latin America and the Caribbean are among the regions with the greatest number of computed tomography scans.

Our study showed a stabilisation of incidence in some Western European countries, confirming findings from the previous study.^38^ An explanation could be the more reserved use of diagnostic activities in those countries. However, it is unlikely that high‐income countries have markedly reduced the use of medical diagnostics, as, for example, within North American regions, imaging rates have continued to increase from 2000 to 2016, albeit at a slower pace in more recent years.^40^ Somewhat more speculative is the explanation that incidence rates have reached their maximum according to the risk factor profile, noting that a further increase in incidence would be less likely even in settings with high diagnostic availability, given decreases in smoking prevalence^26^ and the prospects of stabilising obesity trends.^41^ This explanation does not preclude the possibility that a proportion of kidney cancer cases in regions with greater diagnostic access may constitute overdiagnosis.

Nevertheless, primary prevention offers the greatest potential to reduce kidney cancer incidence globally and should focus on modifiable lifestyle factors. Evidence indicates that smoking cessation,^42^ maintaining a healthy weight through a balanced diet^43^ and regular physical activity^44^ can substantially lower the risk of kidney cancer.

In contrast to kidney cancer incidence rates, mortality has declined in Western Europe, North America, Australia/New Zealand, Japan, South Korea, and Israel, likely reflecting progress in effective curative treatment. To some degree, we can monitor the progress of advanced kidney cancer treatment using outcomes of clinical trials for advanced disease management. In 2000, interleukin‐2 or interferon‐alpha was the primary option, with the median survival of only 11.6 months.^45^ In the following decades, targeted agents, immune checkpoint inhibitors, and up‐front cytoreductive nephrectomy were introduced as options for advanced kidney cancer. A systematic review of clinical trials published in 2023 reported that median survival was 28 months with targeted therapy, which could improve to 40–50 months or more with combinations of ICI and TKI.^46^, ^47^

Disparities in the reduction of kidney cancer mortality could increase with the introduction of new effective but extremely expensive options, such as targeted therapy and immunotherapy. Declines in kidney cancer mortality were not observed in Latin America, Eastern European countries, and the United Kingdom. Latin America was reportedly underrepresented in clinical research, and less than 50% of new cancer drugs launched worldwide between 2009 and 2013 were available in Latin American countries.^48^ In addition, only about half of patients with advanced tumours in clinical trials in Latin America (as well as in Eastern Europe) had nephrectomy before enrolment, highlighting possible variation in access to surgical treatment.^49^ Also, mortality rates did not decrease equally in European countries, probably reflecting similar problems. Important differences in formulary availability, out‐of‐pocket costs, reimbursement, and the availability of many anticancer drugs have been reported in European countries, including those for metastatic kidney cancer.^50^ In contrast to Western Europe, many of the expensive medicines for advanced kidney cancer (e.g., pazopanib, sorafenib, sunitinib, axitinib, and everolimus) were either unavailable or only available at full cost to patients in Eastern Europe.^50^

Our study has several limitations. While the incidence and mortality trends data series available were more comprehensive relative to previous reports, the data were still unavailable for many low‐ and middle‐income countries; specifically, many African and Asian countries were underrepresented in our study.^2^, ^38^ The robustness of IARC's GLOBOCAN estimates varies across countries according to the availability, quality, timeliness, and representativeness of the data^1^, ^19^; low‐resource areas will likely be underrepresented by cancer registries in CI5‐XII and vital statistics.

## 5 CONCLUSION

Our study found significant variations in the incidence of kidney cancer, of which at least some are likely to be explained by known risk factors. Although identifying new risk factors is crucial, more studies should focus on the role of established risk factors, which, combined with access to diagnostic activities, likely explain much of the observed variation. Primary prevention based on information about established cancer risk factors (e.g., smoking and excess body weight) could potentially reduce more than half of the kidney cancer burden.

## AUTHOR CONTRIBUTIONS

**Anton Barchuk:** Conceptualization; methodology; software; project administration; formal analysis; investigation; writing – original draft; visualization; validation; writing – review and editing. **Jerome Vignat:** Methodology; validation; writing – review and editing; software; formal analysis; visualization; data curation. **Kari A. O. Tikkinen:** Validation; writing – review and editing; supervision; methodology. **Ahmedin Jemal:** Validation; writing – review and editing; methodology; supervision. **Freddie Bray:** Supervision; resources; data curation; project administration; formal analysis; conceptualization. **Ariana Znaor:** Investigation; conceptualization; supervision; resources; data curation; writing – review and editing; validation; methodology; project administration.

## FUNDING INFORMATION

Kari A. O. Tikkinen reported funding from the Research Council of Finland (353026), Helsinki University Hospital State Research Funding (TYH2023236), Sigrid Jusélius Foundation, and Vyborg Tuberculosis Foundation.

## CONFLICT OF INTEREST STATEMENT

No commercial entities supported the work reported in the submitted manuscript. No commercial entities provided support that can be viewed as having an interest in the general area of the submitted manuscript. No similar financial associations involve authors' spouses or children. The authors do not have other non‐financial associations relevant to the submitted manuscript.

## Supporting information


**Data S1** Supplementary material.

## Data Availability

The GLOBOCAN 2022 database is available online (https://gco.iarc.fr/en). Further information is available from the corresponding author upon request.
