# Supplementary material for: Global patterns and trends in kidney cancer incidence and mortality
Source: Int J Cancer. 2026 Jan 29;159(1):67–77. doi: 10.1002/ijc.70349 (PMC13140048; doi:10.1002/ijc.70349)
Supplement: Supplementary file 1 — Data S1 Supplementary material. [file IJC-159-67-s001.pdf]

Supplementary material

Global patterns and trends in kidney cancer incidence and mortality
Short title: Global kidney cancer incidence and mortality

Authors: Anton Barchuk, Jerome Vignat, Kari A. O. Tikkinen, Ahmedin Jemal, Freddie Bray, Ariana Znaor

Tables:

1. Table A1: EAPC in kidney cancer incidence and mortality rates in the last 15 and 10 years in countries in Africa, Asia-Pacific, North America, Oceania, Latin America & Caribbean (confidence intervals not crossing null are in bold).
2. Table A2: EAPC in kidney cancer incidence and mortality rates in the last 15 and 10 years in European countries (confidence intervals not crossing null are in bold).

Figures:

1. Figure A1: Kidney cancer incidence (a) and mortality (b) in women in different countries across UN regions
2. Figure A2: Kidney cancer incidence (a) and mortality (b) in women in different countries across UN regions
3. Figure A3: Kidney cancer incidence and mortality trends in women countries in Africa, Northern America, Latin American & the Caribbean, and Oceania
4. Figure A4 Kidney cancer incidence and mortality trends in women in European countries
5. Figure A5: Kidney cancer incidence and mortality trends in men countries in Africa, Northern America, Latin American & the Caribbean, and Oceania
6. Figure A6: Kidney cancer incidence and mortality trends in women in European countries.

Table A1: EAPC in kidney cancer incidence and mortality rates in the last 15 years in countries in Africa, Asia-Pacific, North America, Oceania, Latin America & Caribbean (confidence intervals not crossing null are in bold).

Region	Country	Incidence EAPC (95%CI)	Period	Mortality EAPC (95%CI)	Period
Africa	Uganda	-2.3 (-6.5; 2.1)	2003-2017	-	-
	South Africa	-	-	0.6 (-0.2; 1.3)	2004-2018
Asia	Israel	**-1.5 (-2.1; -0.9)**	2003-2017	**-1.5 (-2.4; -0.6)**	2006-2020
	Philippines	**-1.4 (-2.6; -0.3)**	2003-2017	**1.0 (0.3; 1.7)**	2005-2019
	Bahrain	-1.1 (-5.3; 3.3)	2003-2017	-	-
	Qatar	0.8 (-5.0; 7.0)	2003-2017	-	-
	Kuwait	1.8 (-0.5; 4.2)	2003-2017	-	-

	China	**2.1 (1.3; 2.9)**	2003-2017	-	-
	India	**2.4 (1.5; 3.3)**	2003-2017	-	-
	Thailand	**2.9 (0.9; 4.9)**	2003-2017	-	-
	Korea, Republic of	**4.0 (3.3; 4.7)**	2003-2017	**-1.4 (-2.1; -0.7)**	2007-2021
	Japan	**5.3 (4.9; 5.7)**	2001-2015	**-1.5 (-2.0; -1.0)**	2007-2021
	Armenia	-	-	**-6.6 (-8.5; -4.7)**	2008-2022
	Singapore	-	-	**-2.5 (-3.7; -1.4)**	2008-2022
	Kyrgyzstan	-	-	1.5 (-0.4; 3.5)	2005-2019
	Malaysia	-	-	**4.6 (3.3; 6.0)**	2006-2020
	Venezuela	-	-	**2.3 (1.6; 3.1)**	2002-2016
North America	USA	**0.9 (0.6; 1.3)**	2003-2017	**-1.6 (-1.9; -1.4)**	2007-2021
	Canada	**2.1 (1.7; 2.5)**	2003-2017	**-2.3 (-2.7; -1.9)**	2008-2022
Oceania	New Zealand	**1.2 (0.4; 2.0)**	2003-2017	**-0.9 (-1.5; -0.2)**	2002-2016
	Australia	**2.0 (1.7; 2.3)**	2003-2017	**-2.2 (-2.8; -1.7)**	2008-2022
Latin America & Caribbean	Costa Rica	-0.4 (-1.1; 0.3)	2002-2016	**2.3 (1.1; 3.4)**	2006-2020
	Chile	0.1 (-2.0; 2.2)	2003-2017	**0.9 (0.4; 1.4)**	2007-2021
	Argentina	**1.5 (0.1; 3.0)**	2003-2017	0.4 (-0.1; 0.9)	2007-2021
	Colombia	**3.9 (2.8; 5.0)**	2003-2017	**1.2 (0.5; 1.8)**	2007-2021
	Ecuador	**4.1 (1.9; 6.4)**	2003-2017	-	-
	France, Martinique	**4.3 (1.2; 7.5)**	2003-2017	-	-
	Puerto Rico	**4.8 (4.0; 5.7)**	2003-2017	-2.2 (-4.4; 0.0)	2007-2021
	Nicaragua	-	-	0.1 (-1.6; 1.7)	2007-2021
	Cuba	-	-	0.2 (-0.5; 0.9)	2007-2021
	Guatemala	-	-	0.4 (-1.1; 2.0)	2007-2021
	Mexico	-	-	**0.9 (0.4; 1.4)**	2007-2021
	Brazil	-	-	**1.1 (0.6; 1.6)**	2007-2021
	Uruguay	-	-	**1.3 (0.1; 2.5)**	2006-2020
	Panama	-	-	**1.7 (0.3; 3.1)**	2007-2021
	Paraguay	-	-	**2.3 (0.5; 4.1)**	2007-2021
	Venezuela	-	-	**2.3 (1.6; 3.1)**	2002-2016

Table A2: EAPC in kidney cancer incidence and mortality rates in the last 15 years in European countries (confidence intervals not crossing null are in bold).

**Region**	**Country**	**Incidence EAPC (95%CI)**	**Period**	**Mortality EAPC (95%CI)**	**Period**
Europe	Austria	**-0.9 (-1.3; -0.5)**	2003-2017	**-2.8 (-3.3; -2.2)**	2007-2021
	Czechia	**-0.4 (-0.7; -0.1)**	2003-2017	**-3.1 (-3.5; -2.7)**	2007-2021
	Iceland	0.0 (-1.6; 1.5)	2007-2021	-	-
	Germany	0.2 (0.0; 0.5)	2003-2017	**-2.0 (-2.4; -1.5)**	2006-2020
	Lithuania	0.3 (-0.4; 1.0)	2003-2017	**-2.3 (-3.3; -1.2)**	2008-2022
	Poland	0.7 (-0.5; 2.0)	2003-2017	**-2.6 (-3.0; -2.2)**	2007-2021
	Switzerland	0.7 (0.0; 1.5)	2003-2017	**-2.8 (-3.5; -2.1)**	2007-2021

	Estonia	0.8 (-0.1; 1.6)	2003-2017	**-2.5 (-4.0; -1.0)**	2008-2022
	Finland	**0.8 (0.3; 1.3)**	2007-2021	**-1.9 (-2.6; -1.3)**	2007-2021
	Sweden	**0.9 (0.1; 1.6)**	2007-2021	**-3.0 (-3.6; -2.5)**	2008-2022
	Slovenia	0.9 (0.0; 1.9)	2003-2017	-1.2 (-2.5; 0.1)	2006-2020
	Norway	**1.0 (0.4; 1.7)**	2007-2021	-1.0 (-2.2; 0.1)	2002-2016
	The Netherlands	**1.3 (0.8; 1.8)**	2003-2017	**-3.1 (-3.7; -2.6)**	2008-2022
	France (metropolitan)	**1.6 (1.3; 1.9)**	2003-2017	**-1.3 (-1.9; -0.7)**	2006-2020
	Italy	**2.0 (1.4; 2.5)**	2003-2017	**-0.9 (-1.2; -0.5)**	2006-2020
	Denmark	**2.1 (1.6; 2.5)**	2007-2021	**-3.7 (-4.9; -2.5)**	2007-2021
	Croatia	**2.3 (1.6; 3.0)**	2003-2017	-0.3 (-1.1; 0.6)	2007-2021
	Latvia	**2.3 (1.5; 3.2)**	2003-2017	**-1.6 (-2.2; -1.1)**	2007-2021
	Ireland	**2.4 (1.7; 3.0)**	2003-2017	**-2.0 (-3.5; -0.6)**	2006-2020
	Spain	**2.4 (1.9; 2.9)**	2002-2016	-0.4 (-0.9; 0.0)	2007-2021
	Belarus	**2.7 (2.2; 3.1)**	2003-2017	-	-
	Türkiye	**3.1 (2.3; 4.0)**	2003-2017	-	-
	Cyprus	**3.3 (1.5; 5.1)**	2003-2017	-	-
	UK	**3.6 (3.2; 3.9)**	2003-2017	**-0.9 (-1.1; -0.6)**	2006-2020
	Malta	**6.1 (4.4; 7.9)**	2003-2017	-	-
	Belgium	-	-	**-2.4 (-3.2; -1.7)**	2006-2020
	North Macedonia	-	-	-2.3 (-4.7; 0.2)	2007-2021
	Slovakia	-	-	**-1.6 (-2.7; -0.4)**	2007-2021
	Hungary	-	-	**-0.7 (-1.2; -0.2)**	2008-2022
	Serbia	-	-	-0.6 (-1.4; 0.2)	2008-2022
	Greece	-	-	0.2 (-0.6; 1.1)	2006-2020
	Bulgaria	-	-	0.5 (-0.3; 1.3)	2007-2021
	Portugal	-	-	**1.4 (0.8; 2.0)**	2005-2019
	Romania	-	-	**1.6 (1.0; 2.2)**	2005-2019
	Moldova	-	-	**1.8 (0.3; 3.3)**	2004-2018

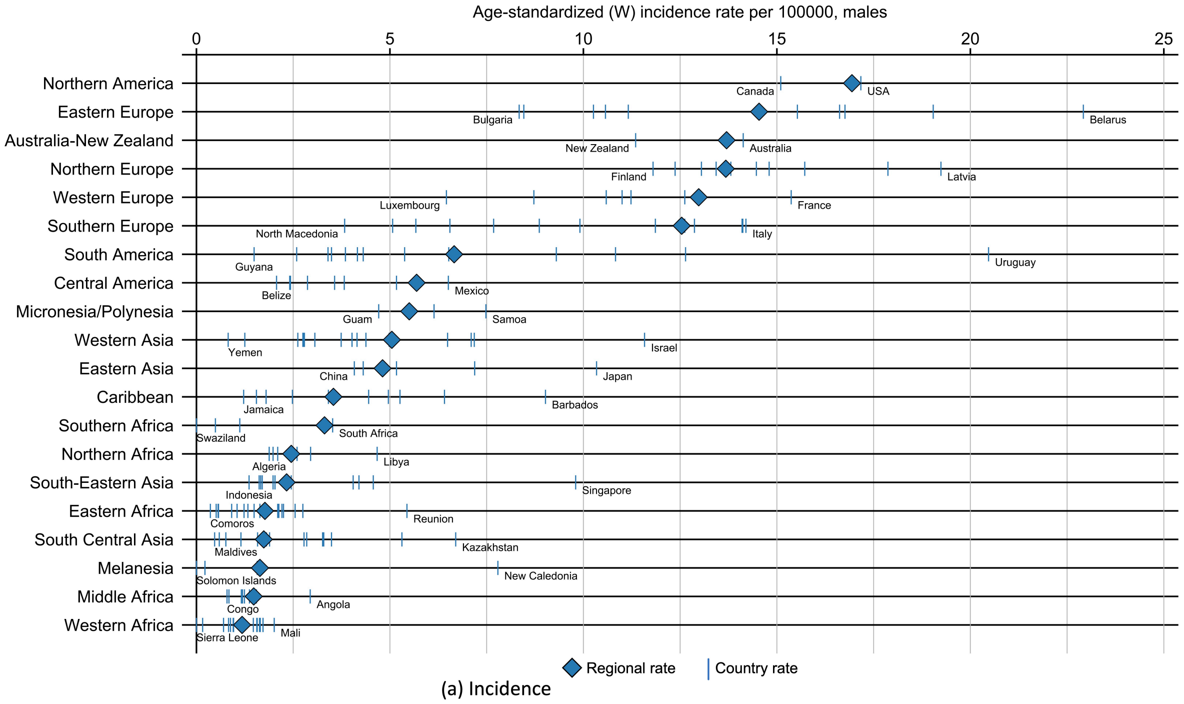

(a) Incidence

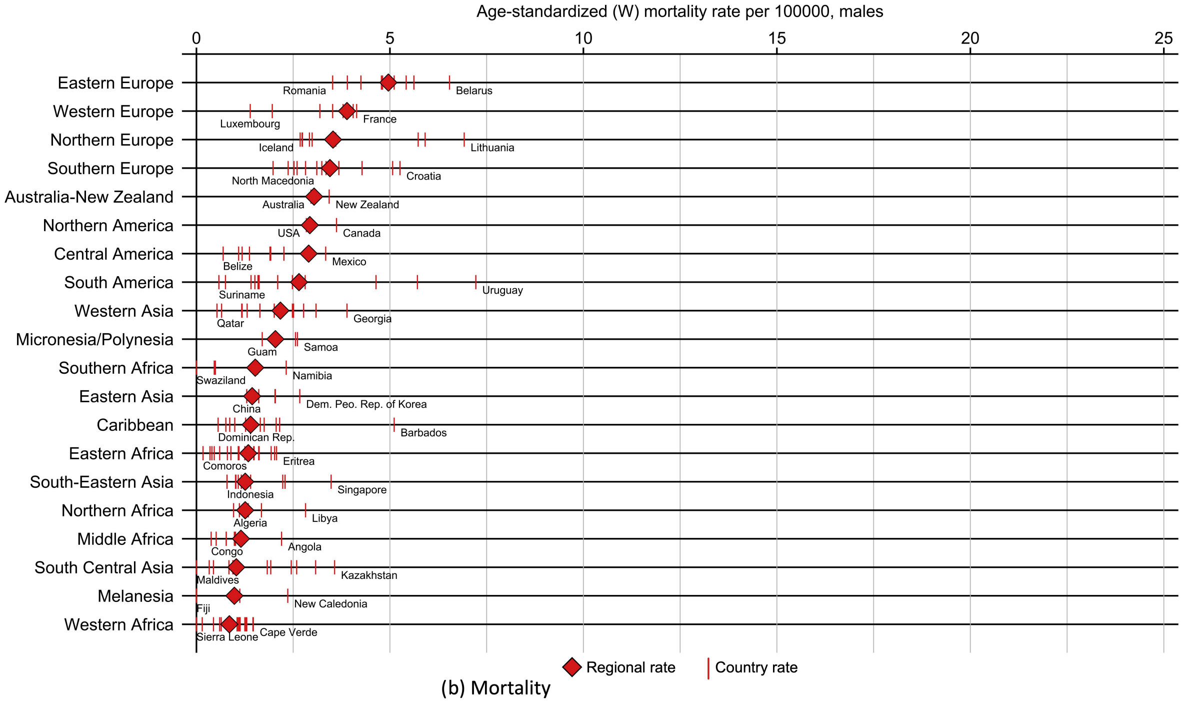

(b) Mortality

Figure A1: Kidney cancer incidence (a) and mortality (b) in men in different countries across UN regions

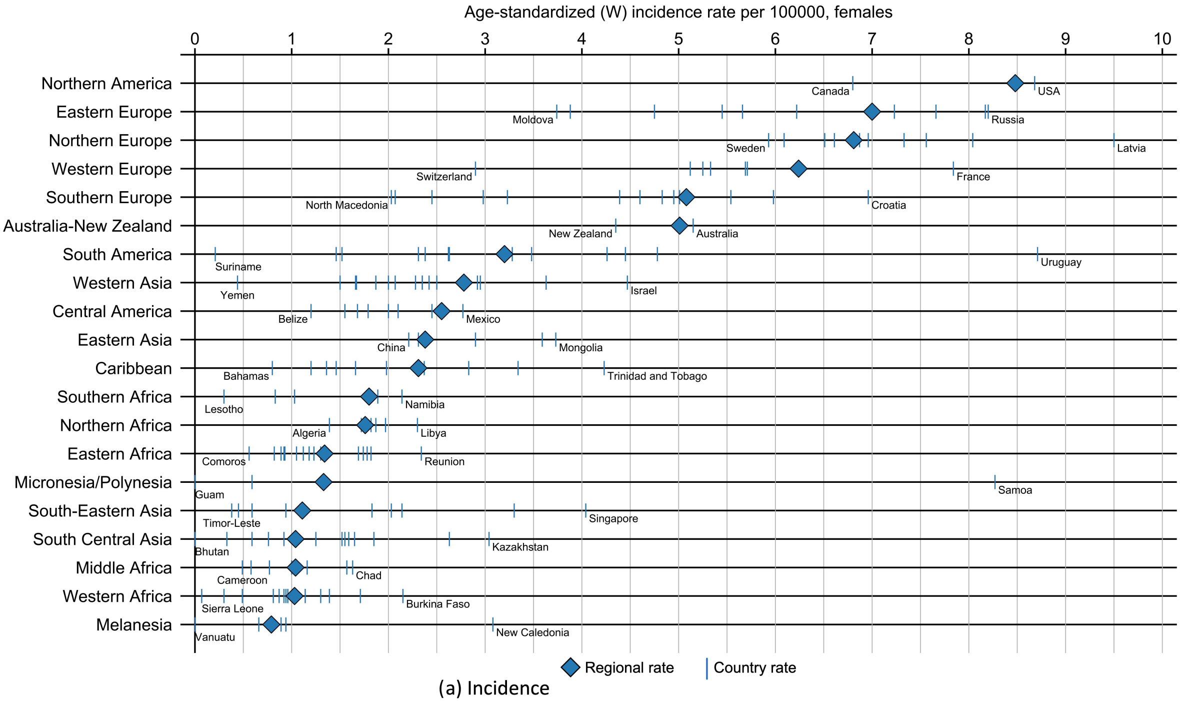

(a) Incidence

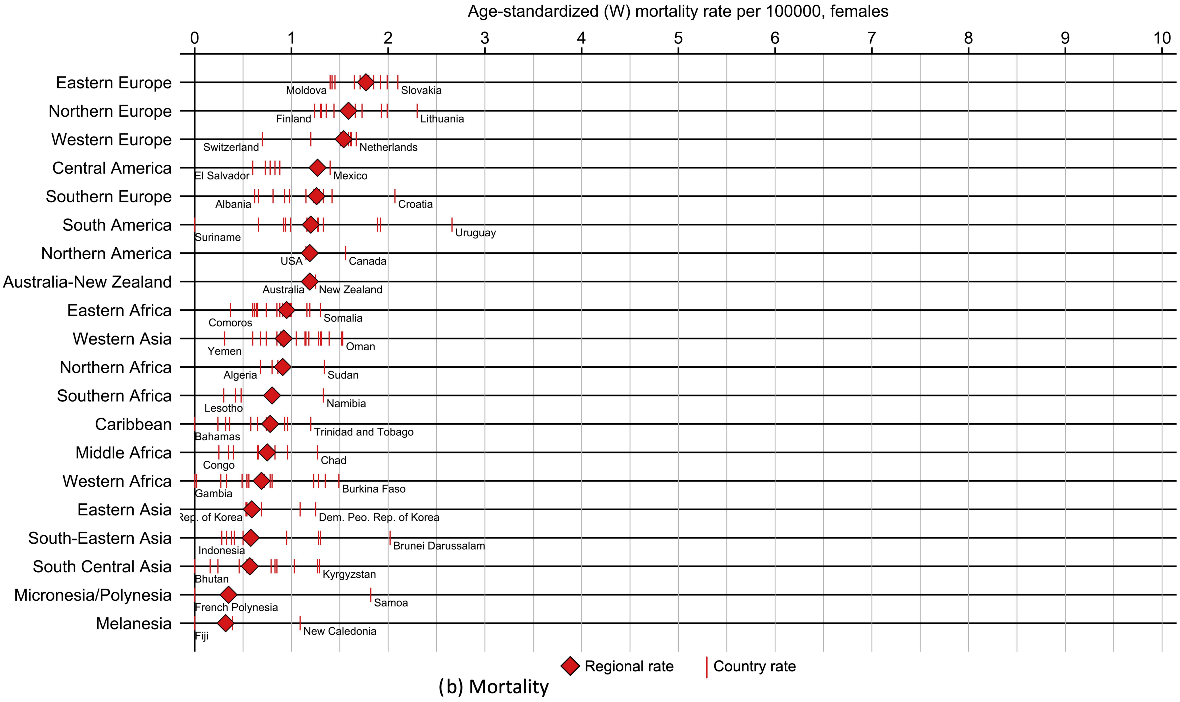

(b) Mortality

Figure A2: Kidney cancer incidence (a) and mortality (b) in women in different countries across UN regions

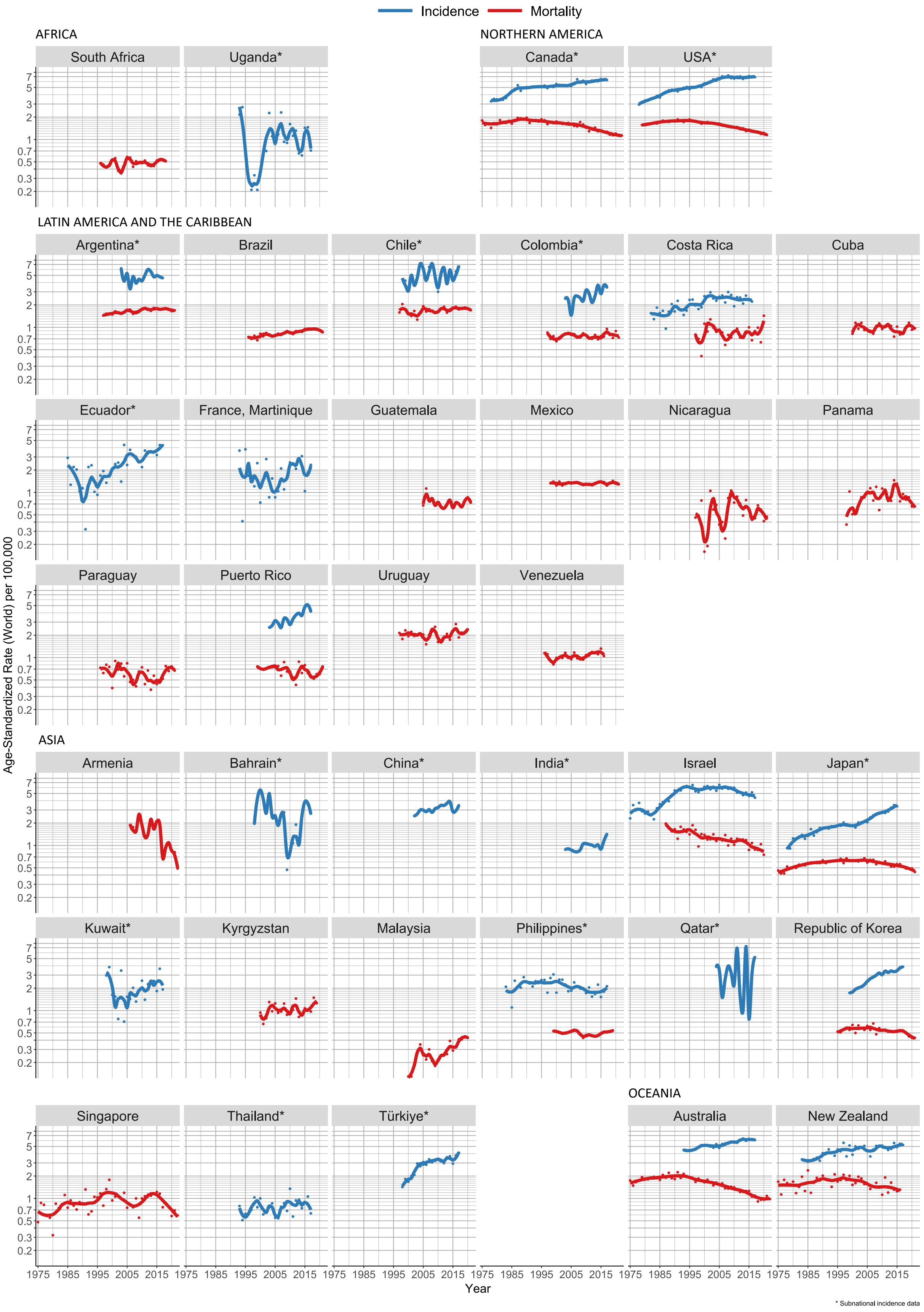

Figure A3: Kidney cancer incidence and mortality trends in women countries in Africa, Northern America, Latin American & the Caribbean, and Oceania

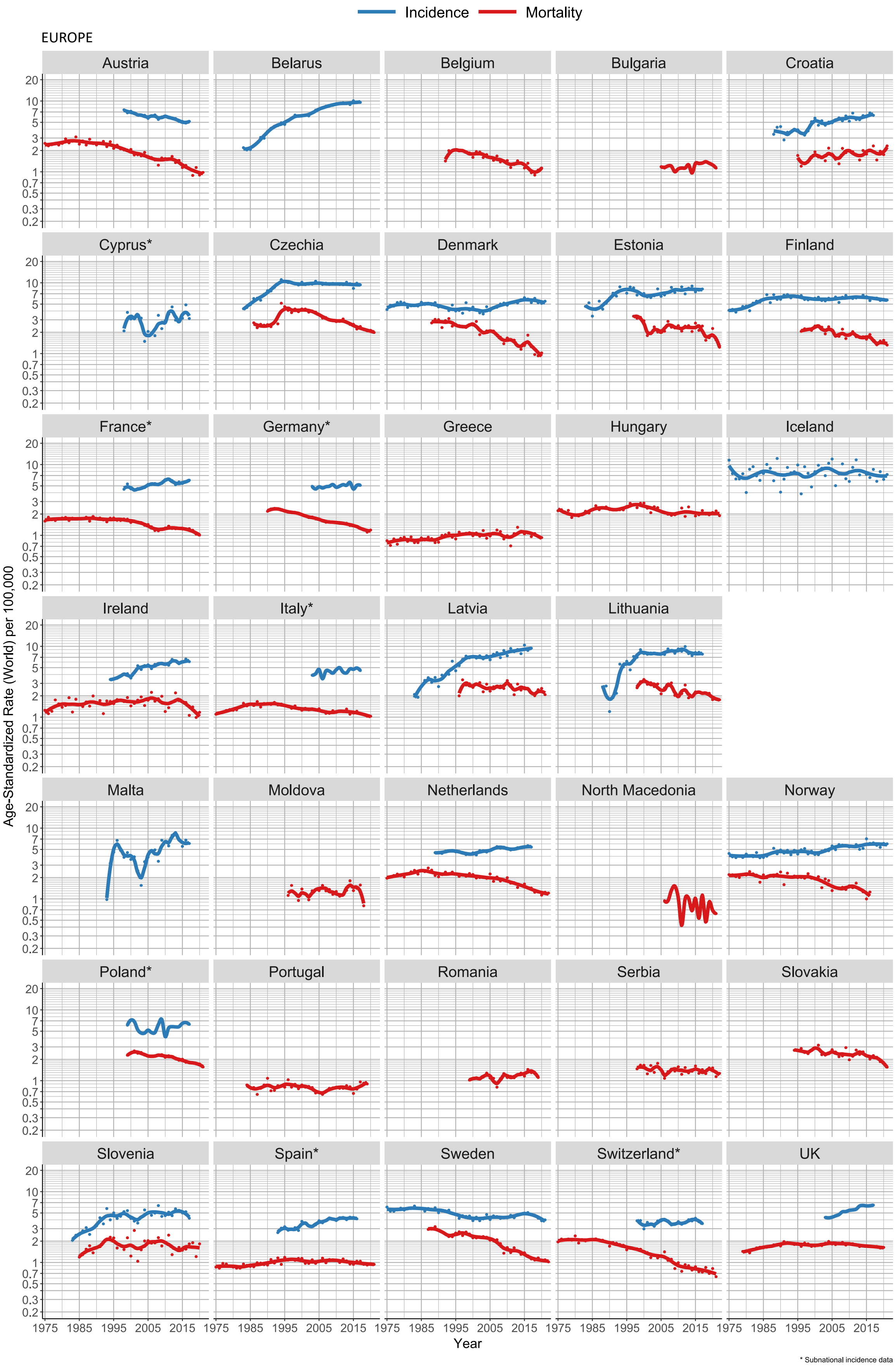

Figure A4: Kidney cancer incidence and mortality trends in women in European countries.

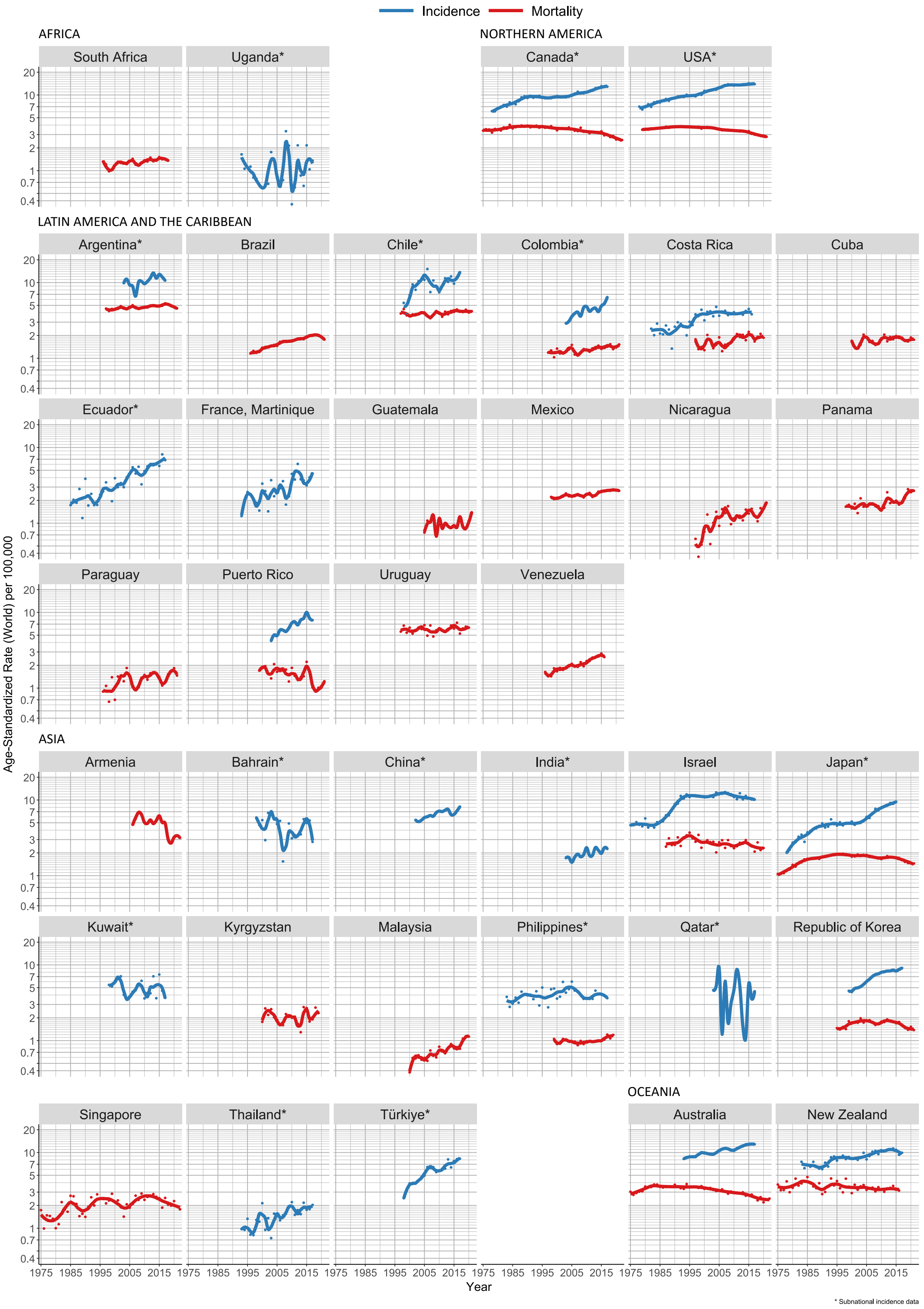

Figure A5: Kidney cancer incidence and mortality trends in men countries in Africa, Northern America, Latin American & the Caribbean, and Oceania

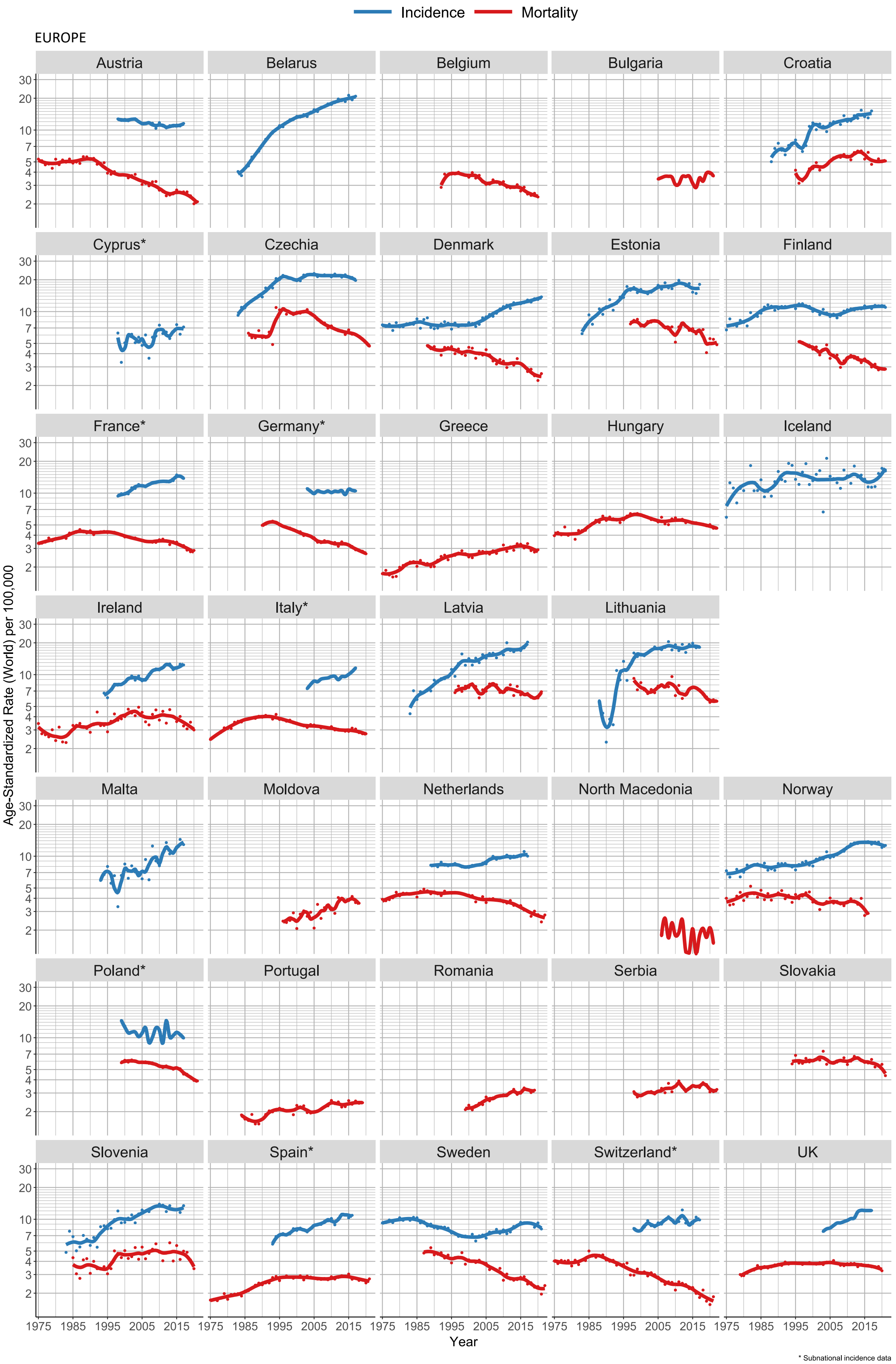

Figure A6 Kidney cancer incidence and mortality trends in men in European countries.
